# Quantification of Hair Corticosterone, DHEA and Testosterone as a Potential Tool for Welfare Assessment in Male Laboratory Mice

**DOI:** 10.3390/ani10122408

**Published:** 2020-12-16

**Authors:** Alberto Elmi, Viola Galligioni, Nadia Govoni, Martina Bertocchi, Camilla Aniballi, Maria Laura Bacci, José M. Sánchez-Morgado, Domenico Ventrella

**Affiliations:** 1 Department of Veterinary Medical Sciences, University of Bologna, 40064 Ozzano dell’Emilia, BO, Italy; alberto.elmi2@unibo.it (A.E.); nadia.govoni@unibo.it (N.G.); martina.bertocchi3@unibo.it (M.B.); camilla.aniballi2@unibo.it (C.A.); marialaura.bacci@unibo.it (M.L.B.); 2 Comparative Medicine Unit, Trinity College Dublin, D02 Dublin, Ireland; GALLIGIV@tcd.ie (V.G.); jose.sanchez-morgado@tcd.ie (J.M.S.-M.)

**Keywords:** hair steroids, male mice, laboratory animals, corticosterone, testosterone, DHEA, age, housing

## Abstract

**Simple Summary:**

Mice is the most used species in the biomedical research laboratory setting. Scientists are constantly striving to find new tools to assess their welfare, in order to ameliorate husbandry conditions, leading to a better life and scientific data. Steroid hormones can provide information regarding different behavioral tracts of laboratory animals but their quantification often require stressful sampling procedures. Hair represents a good, less invasive, alternative in such scenario and is also indicative of longer timespan due to hormones’ accumulation. The aim of the work was to quantify steroid hormones in the hair of male laboratory mice and to look for differences imputable to age and housing conditions (pairs VS groups). Age influenced all analysed hormones by increasing testosterone and dehydroepiandrosterone (DHEA) levels and decreasing corticosterone. When comparing animals housed in pairs VS groups, the only difference found was a higher level of DHEA in mice housed in groups. Due to the nature of DHEA, this finding may suggest that group housing may be beneficial for social interaction. In conclusion, it seems that hair hormones quantification may be a good tool for welfare assessment in laboratory mice and may help in refining husbandry.

**Abstract:**

Steroids, providing information regarding several biological patterns including stress and sexual behavior, have been investigated in different matrices in laboratory mice. Data regarding hair quantification, indicative of longer timespans when compared to blood and saliva, are lacking. The aim of the work was to analyze the hormonal hair profile of laboratory male mice and to investigate potential relationships with age and housing, as a potential tool for welfare assessment. Fifty-six adult male C57BL/6J and C57BL/6OlaHsd substrain mice were included in the study, housed in pairs or groups. Testosterone (T) and dehydroepiandrosterone (DHEA) were quantified by radioimmunoassay, corticosterone (CORT) by ELISA. Mean hormone levels were 6.42 pg/mg for T, 23.16 pg/mg for DHEA and 502.1 pg/mg for CORT. Age influenced all hormones by significantly increasing T and DHEA levels and decreasing CORT; only DHEA, significantly higher in grouped mice, was influenced by housing conditions. The influence of age indicates the need for accurate age-related reference intervals, while the higher levels of DHEA in grouped animals suggests that such housing practice may be beneficial for social interactions. In conclusion, it seems that hair hormones quantification may be a good tool for welfare assessment in laboratory mice and may help in refining husbandry.

## 1. Introduction

The laboratory mouse, derived from the wild mouse (*Mus musculus*), is the most used animal species in the biomedical research field [1,2]. When in their natural environment, mice exhibit a complex social organization consisting in small groups composed by one adult male, several female and their offspring, occupying a territory. The adult, sexually mature male is territorial, competitive and aggressive with intruders. Laboratory mice, inbred for many generations, still continue to express such species-specific social behavior despite decades of human influence [3,4,5]. In this species, as for many others, the behavioral pattern, thus the state of welfare of the animal, is intimately connected and influenced by the hormonal profile [6,7,8]. The Hypothalamic-Pituitary-Gonadal (HPG) and Hypothalamic-Pituitary-Adrenal (HPA) axis are the main pathways responsible for behavioral modulation. The pivotal hormones of these axis are steroids, synthetized starting from cholesterol mainly at the level of gonads and adrenal gland [9]. In mice, steroids have mainly been assessed on several matrices such as plasma [9,10,11,12,13], urine [14,15,16], feces [17,18] and saliva [19]. Nonetheless, lately, hair steroids have been studied and analyzed in numerous species [20,21,22,23,24] including humans [25,26] and rodents [27], the latter both in wild [28] and laboratory settings [29,30,31]. The more classical matrices blood and saliva are indicative of a short time period and are strongly influenced by the peculiar daily fluctuations of such hormones; on the other hand, hair is capable of providing medium/long-term retrospective information and overcomes the problem of repeated sampling necessary for an integrated steroid response [32]. The levels of hormones accumulated in the hair shaft are necessarily related to the species-specific hair follicle cycle: in mice it consists of 14 days of growth followed by 14 days of rest, with a highly synchronization of neighboring hair, reflecting up to three/four months [28].

Corticosterone (CORT) is considered as the main glucocorticoid in rodents, as opposite to other mammals where cortisol is predominant and is involved in different physiological or stressful conditions [33,34]. Despite plasma levels of cortisol and corticosterone being closely correlated in mice, with both hormones being interchangeably used as indicators of stress, CORT has been identified as the best adaptation-related biomarker of chronic stress in this species [10]. Testosterone (T) is instead the main sexual androgen in male mice and it is associated to the reproductive cycle; it is also involved in the modulation of aggressive behavior acting on androgen and estrogen receptors in the male brain [11,12,35]. Plasmatic levels of T are age-related, with a peak between 30 and 60 days of life (puberty) and a slow decrease thereafter, with a circadian pulsatile secretion [6,12]. Moreover, T is affected by stress, indeed it has been proven how stressors can induce a rapid increase in its levels [36,37]. Dehydroepiandrosterone (DHEA) is a polyvalent hormone with various functions. Generally speaking, it is an androgens precursor and, in humans, it is one of the highest circulating steroid synthetized by the adrenal glands. Nonetheless, it can also be synthetized and metabolized by gonads and the brain itself [38]. In rodents, quite peculiarly, DHEA cannot be produced by the adrenals due to a local enzymatic deficit of p450c17. Moreover, when it comes to T production in this species, the predominant pathway is the Δ4 one, which uses progesterone and not DHEA as precursor. In any case, rats and mice show higher concentrations of DHEA in the brain when compared to plasma [39]. Evidence suggests that DHEA, in addition to its role as sexual hormone, also has neuroprotective and anti-glucorticoids effects, reducing aggressive behavior [13,39,40,41]. In the light of the above-mentioned reasons, it seems that evaluating such hormonal panel in a non-invasive matrix as hair, may represent a good tool to assess environmental-related welfare in mice housed in experimental facilities, in full respect of the current legislation for the use of animal in biomedical research. Moreover, being capable of quickly assessing stress thus correct potentially wrong environmental conditions, would lead to more repeatable and robust scientific outcomes.

## 2. Materials and Methods

### 2.1. Animals and Husbandry

Fifty-six sexually mature male mice (*n* = 56) of C57BL/6J and C57BL/6OlaHsd substrains, bred at the Comparative Medicine Unit of Trinity College Dublin were included in the present study. Management and husbandry procedures were in accordance with S.I. 543 of 2012 [42], 2010/63/EU [43] and 2007/526/EC [44]. Animals were fed with irradiated PicoLab Rodent Diet 20 5053 (LabDiet, St. Louis MO, US), received ad libitum reverse osmosis water and were exposed to 12/12 hours light/dark cycle. Health monitoring was performed according to the Federation of European Laboratory Animal Science Associations (FELASA) recommendations [45] and the facility was negative to all agents included in the list. All animals were born in the facility and, upon weaning (21–25 days of life), were either grouped in large cages (Tecniplast 1500U cage; Tecniplast spa, Buguggiate, Varese, Italy) of 15–20 mice or paired for future breeding, as brother-sister matings, in smaller cages (Tecniplast GM500 cage; Tecniplast spa, Buguggiate, Varese, Italy). All cages were bedded with poplar wood (SAFE Select, SAFE-lab, Rosenberg, Germany); nesting material was Bed-r’Nest (Datesand, Manchester, UK). Animals were handled by picking them up using red polycarbonate tunnels (Serlab, London, UK) or the open hand [46,47,48]. All animals included in the present studies were either untreated controls from other experimental studies or breeders at the end of their reproductive cycle. All experimental procedures were approved by Trinity’s Animal Research Ethics Committee (AREC) and authorised by the Irish Health Products Regulatory Authority (HPRA) and in accordance with the relevant Irish and European regulations [42,43]. Such opportunist samplings are in accordance with the 3R principles.

### 2.2. Hair Sampling and Steroids Extraction

Hair was trimmed for its full length from the back area of each mouse, more specifically from the neck to the base of the tail, with an electric razor on a dead animal following one of the Annex IV methods of humane killing [43]. Samples placed in individual biohazard transport bags were stored at room temperature, in dark conditions to avoid light exposure and shipped to the Department of Veterinary Medical Sciences of the University of Bologna for the analyses.

Steroid extraction was performed as previously described by Bacci et al., 2014 [20], with slight modifications. Briefly, 100 mg of each sample were washed with deionized water and isopropanol (American Chemical Society grade, Carlo ERBA Reagents srl, Milano, Italy) in order to remove any organic residue from the surface. Once fully dried, samples were cut with scissors and a scalpel blade until they were finely pulverized. For corticosterone extraction, 2 mL of methanol (HPLC grade, Carlo ERBA Reagents srl, Milano, Italy) were added to 10 mg of powdered hair and incubated overnight in rotation (Roller mixer SRT6, Stuart Equipment, UK). After centrifugation at 10,000× *g* for 30 min, 0.6 mL of methanol were collected and evaporated to dryness under an air-stream suction hood. Dried extracts were reconstituted with 250 µL of the dedicated kit assay buffer (see following section for details) and immediately analyzed. For testosterone and DHEA extraction, 60 mg of powdered hair were incubated overnight in rotation with 3 mL of methanol. After centrifugation at the same afore-mentioned conditions, 2 mL of methanol were collected, evaporated to dryness under an air-stream suction hood and stored at −20 °C until analysis.

### 2.3. Corticosterone Quantification by ELISA

Corticosterone was measured by means of a commercial competitive immunoassay (Corticosterone Competitive ELISA kit, Thermo Fisher Scientific, Life Technologies Corporation, MD, USA) following the manufacturer’s instructions. Briefly, 50 µL of either each standard (range 78.12–1250 pg/mL) or each sample were loaded in duplicate in a microtiter plate coated with anti-sheep IgG and added with 25 µL of corticosterone conjugate and 25 µL of corticosterone antibody; a 1 h incubation at room temperature with shaking followed. After four washes, 100 µL of tetramethylbenzidine (TMB) substrate were loaded into the wells and incubated for further 30 min. After incubation, 50 µL of stop solution were added and adsorbance was measured using a microplate reader (Infinite F50, Tecan, Grodig, Austria) at 450 nm within 10 min.

The cross-reactivity of the antibody was: Corticosterone 100%, Desoxycorticosterone 12.3%, Tetrahydrocorticosterone 0.76%, Aldosterone 0.62%, Cortisol 0.38%, Progesterone 0.24%, Dexamethasone 0.12%, Cortisone and Estradiol < 0.08%.

In order to determine the parallelism between hormone standards and endogenous hormone in mouse hair, a pooled sample containing high concentrations in corticosterone was serially diluted (1:1–1:8) with assay buffer and used for a simple linear regression analysis. A good parallelism was confirmed by the outcoming *r*^2^ of 0.97. Precision was estimated by repeatedly extracting and assaying samples in the same assay (4 times) and the mean intra-assay coefficient of variation was 4.05%. Reproducibility was estimated by assaying 3 samples in independent assays and the mean inter-assay coefficient of variation was 9.9%. The analytical sensitivity of the assay was 32.4 pg/well.

### 2.4. Testosterone and DHEA Analysis by Radioimmunoassay (RIA)

The dry extracts were reconstituted in assay buffer for measurement of testosterone (8 mg hair equivalent) or DHEA (4 mg hair equivalent) by radioimmunoassay; tritiated testosterone (30 pg/tube; 83.4 Ci/mmol; PerkinElmer inc. Boston, MA, USA) or tritiated DHEA (30 pg/tube; 76.1 Ci/mmol PerkinElmer inc. Boston, MA, USA) were added, followed by rabbit anti-testosterone serum (0.1 mL, 1:50,000; produced in our laboratory) or rabbit anti-DHEA serum (0.1 mL, 1:10,000; produced in our laboratory), respectively. After incubation and separation of antibody-bound and -unbound steroid by charcoal-dextran solution (charcoal 0.25%, dextran 0.02% in phosphate buffer), tubes were centrifuged (15 min, 3000× *g)*, the supernatant was decanted and radioactivity immediately measured using a β-scintillation counter (Packard C1600, Perkin Elmer, USA).

The sensitivity of the testosterone assay was 2.80 pg/tube and the intra- and inter-assay coefficients of variation were 5.7% and 8.7% respectively. The sensitivity of the DHEA assay was 1.91 pg/tube and the intra- and inter-assay coefficients of variation were 4.9% and 10.5% respectively. Cross reactions of various steroids with antiserum raised against testosterone were: testosterone (100%), dihydrotestosterone (25.44%), androstenedione (0.6%), progesterone and cortisol (<0.0001%). Cross reactions of various steroids with antiserum raised against DHEA were: dehydroepiandrosterone (100%), dehydroepiandrosterone sulfate (39%), androstenedione (10%), testosterone (0.25%), progesterone and cortisol (<0.001%). In order to determine the parallelism between hormone standards and endogenous hormones in mouse hair, a pool sample containing high concentrations in DHEA or testosterone was serially diluted (1:1–1:8) with assay buffer and used for a regression analysis. A good degree of parallelism was confirmed by the outcoming *r*^2^ of 0.97). The assay results for steroids hormones were expressed as pg/mg of hair.

### 2.5. Statistical Analyses

Additional data recorded for each animal included age (days) and type of housing (pair vs group). All statistical analyses were performed using the software R 3.6.3 (The R Foundation for Statistical Computing, https://www.r-project.org/) and graphically represented using the software GraphPad Prism v.8 (GraphPad Software Inc., San Diego, CA, USA). Descriptive statistics were calculated and reported as mean ± SD. Normal distribution and equality of variance were tested by means of Shapiro-Wilk and Levene tests respectively. For each hormone, potential differences imputable to the substrains were assessed by means of non-parametric Mann-Whitney *U* tests. Spearman rank correlation test was used to assess relationships between steroids and age. In order to assess the effect of age on hormones’ concentrations, linear regression models were set up. For this purpose, if normal distribution was rejected, logarithmic transformations were applied and normality was re-tested as previously reported. Diagnostics of models were performed testing normal distribution of residuals (Shapiro-Wilk test) and evaluating them in graphical distribution. In order to evaluate differences in hormonal profiles imputable to housing conditions (pairs VS groups), 17 animals were selected, taking into account ages in order to avoid biases and divided into two groups (pairs *n* = 7 and mean age of 140 days; groups *n* = 10 and mean age of 132 days). Either student’s *t* test or Welch test were used to evaluate differences between the two groups. The significance level for all statistical analyses was set at *p* < 0.05.

## 3. Results

Fifty-six male mice were evaluated in the present study with mean age of 253 ± 90.9 days. Animals did not show any lesion imputable to pathologic processes nor behavioural alterations. Mean hormone levels were: 6.42 ± 1.84 pg/mg of hair for testosterone (T), 23.16 ± 7.00 pg/mg of hair for DHEA and 502.1 ± 197.7 pg/mg of hair for corticosterone (CORT). For all analysed hormones, no differences were recorded between the two substrains.

The correlation coefficients (ρ) resulting from the Spearman correlation rank test are reported in Table 1.

All steroids were statistically correlated to each other; in particular CORT resulted inversely correlated to both T (ρ = −0.587; *p* < 0.0001) and DHEA (ρ = −0.479; *p* = 0.0003), while T and DHEA showed a very strong positive correlation (ρ = 0.855; *p* < 0.0001). As for age, it was strongly inversely correlated to CORT (ρ = −0.604; *p* < 0.0001) and mildly positively correlated to T (ρ = 0.451; *p* = 0.0006) and DHEA (ρ = 0.354; *p* = 0.0086).

**Figure 1:**
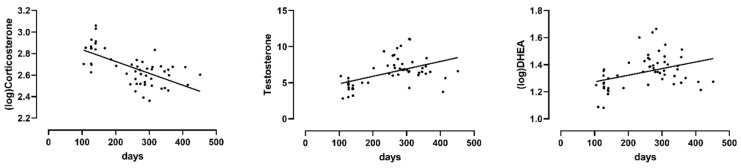
Linear regression model between the three hair hormone concentration (pg/mg) and age (days).

Logarithmic transformations were necessary for CORT and DHEA for the linear regression models carried out to evaluate the effect of age on hormones’ concentrations; age was always statistically significant (*p* < 0.001). The angular coefficients (β) are reported in Table 2, while the graphical results of the linear regression models are presented in Figure 1.

**Figure 2:**
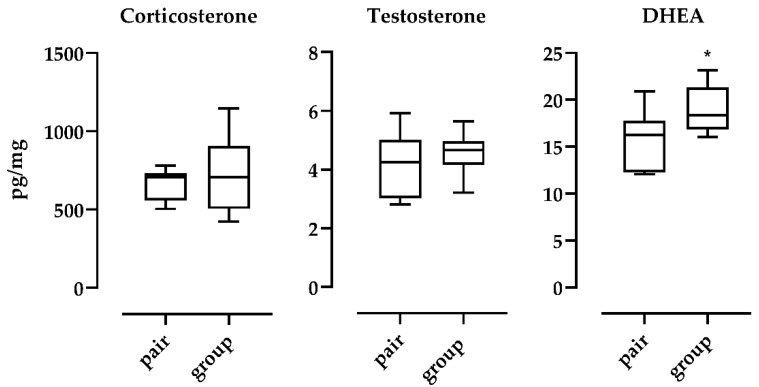
Comparison between housing conditions (pair *n* = 7 vs. groups *n* =10) for the analysed steroids. *: *p* < 0.05.

Comparisons between groups according to housing conditions (pairs VS groups) are represented in Figure 2. The concentration of DHEA was statistically affected by the type of housing (*p* = 0.0377, Student’s *t* test) while CORT (*p* = 0.3979, Welch test) and T (*p* = 0.5446, Student’s *t* test) remained relatively stable; hair CORT levels in male mice housed in groups were more variable.

## 4. Discussion

The aim of the work was to analyze the hormonal hair profile of laboratory mice and to investigate potential relationships with age and housing. Indeed, it is very well acknowledged how steroids can reflect the welfare status of animals, which is influenced by the above-mentioned parameters [8]. Looking for good and reliable welfare indicators for laboratory mice is necessary to improve animals housing and management in respect of both International legislations and the 3Rs principle [49]. Moreover, information regarding the steroid profiles of male mice at different ages and under different housing conditions, may provide better knowledge and characterization of murine models, leading to more robust and standardized experimental data [50]. Hormones hereby quantified and analyzed are involved with both stress (via HPA) and sexual behavior (via HPG): corticosterone, testosterone and dehydroepiandrosterone. Regarding the chosen biological matrix, hair was selected as non-invasive to collect and relatively stable.

In the murine species, steroids assessment in hair was previously reported in a very small number of studies and using different methodologies [28,29,30,31,51]. Overall, the results of the quantifications reported in this study are in agreement with existing literature, with the only exception of CORT that shows higher values when compared to some of the previous studies. Such difference may be explained by a wide variety of factors such as the study design, in terms of age of the animal and grouping factor and the chosen methodology. Nonetheless, the results still seem to be robust and reliable as suggested by the good inter- and intra-assay coefficients of the ELISA kit.

It is very well acquainted that different mouse strains lead to different behavioral patterns, especially when it comes to aggression [52]. Since animals in the present studies belonged to the same strain but difference substrains (C57BL/6J and C57BL/6OlaHsd) a comparative analysis was performed. No differences in hair hormones concentrations were recorded.

When it comes to analyzing the effects of age on the hair hormonal profile, literature is even scarcer. Carlitz et al. [28] reported how, in wild mice, hair steroid concentrations, just like plasmatic levels, were influenced by age, with higher levels of TEST and CORT in older animals. Based on the hereby reported results, this finding was only partially confirmed in laboratory mice; indeed, despite TEST being again higher in older animals, CORT levels showed an opposite trend. According to the correlation analysis, both hormones were correlated with age either positively (TEST) or negatively (CORT) but only CORT in a statistically significant manner. Explaining the TEST increase, consistent for both wild and laboratory mice, is relatively easy, as the key reason is most likely linked to puberty and sexual maturation, occurring during the second month of life [6]. When it comes to CORT, the higher levels found in younger laboratory mice when compared to older ones, may be related to the fact that pups are often handled for routine husbandry such as sex confirmation and cage allocation and slowly get used to human interactions as they get older. Such hypothesis seems to be strengthen by the work of Mucignat-Caretta and colleagues, showing how young mice are more responsive to mild stressors, such as environmental ones, with higher CORT plasmatic peaks [13]. The animals used in the present study were handled using tunnels and open hands, rather than being picked up by the tails. Several papers have already proven how this procedure allows for fast habituation thus lowering the stress levels, mainly on the basis of behavioral investigations [47,48].Comparing CORT levels of young mice handled with and without tunnels may provide interesting insight to such habituation process. As previously stated, the decreasing CORT trend is opposed to the one described for wild mice [28]. This may be because, in the wild, sexually mature adult male mice have to face a wide range of stressing situations related to territoriality and hierarchy and are constantly prey for other animals. As for DHEA, to the best of the authors’ knowledge, no data are available in literature regarding its hair levels in relation to age in male mice. The results of the present study show an increasing trend overtime, as for the other androgen TEST, yet very weak according to both statistical approaches. In humans, plasmatic levels of DHEA peak during the third decade of life and slowly decrease thereafter [53]; a similar situation has been reported also in mice, with lower plasmatic levels in old animals [54]. The physiological temporal delay between plasmatic peak and hair accumulation may explain the increasing trend of DHEA hair levels. Nonetheless, it is still important to acknowledge that the regression line shows a very mild inclination, potentially suggesting, also in this case, a decrease in plasmatic levels overtime thus lower hair accumulation. Overall, this study highlights the importance of considering age as a key factor when analyzing steroids, especially in a “long-term” indicative matrix such as hair and the need for age-related physiological ranges.

Age being equal, the housing condition only seemed to induce differences in DHEA levels, with higher values in male mice housed in groups. As for the other two hormones, no differences were highlighted confirming what has been already reported by other studies [55]; still it can be noted how CORT showed higher variability in grouped animals while TEST in paired ones. Generally speaking, hair CORT represents the average trend of its plasmatic concentrations, thus providing long term information about the HPA activation trends and potential underlying para-physiological and pathological statuses. Extended “chronic” stressing situations, as social instability and behavioral alterations, are capable of leading to higher hair CORT levels as previously reported for female mice [29]. On the other hand, acute yet repeated stressing events as anesthesia do not seem to lead to higher hair CORT levels [31]. When it comes to pathological conditions, it has been reported how murine models of diabetes mellitus show higher hair CORT levels upon symptoms presentations [30]. Housing male mice in groups can be a delicate practice as it can potentially lead to aggressiveness and sometimes death, but, since mice are social animals, groups seem to better respect their ethological needs. In order to avoid problems and reduce stress, the best moment to group male mice is right after weaning, when animals are still young and hierarchy is still to be established [4]. This hypothesis is confirmed by the results of the present study as hair CORT of male mice grouped immediately after weaning was not different when compared to paired animals. The high variability in hair CORT levels of grouped mice can be imputed to the fact that, as in the wild, every group has its own hierarchy with some dominant and some submissive animals. Nonetheless, despotic hierarchies can be ruled out for the animals enrolled in the present study, as this would have led, according to literature, to alterations in TEST levels [56]. As already stated, only hair DHEA was significantly influenced by the different type of housing, with higher levels in grouped animals. T and CORT being equal, this finding may be indicative of higher social stimulation and interaction, again supporting the better ethological value of grouping, as DHEA acts as a neuromodulator and anti-glucocorticoid agent. Nonetheless, due to its plethora of biological roles, some of which still partially unknown, it is not possible to formulate a definitive hypothesis. Overall, what can be certainly stated is that the quantification of one steroid itself is not sufficient to have a clear view of the welfare status of the animals. Analyzing panels of different hormones, often related to each other, helps widening the point of view and better understanding the biology of the used model.

It is also important to acknowledge that being able to perform repeated measurements of hair hormones may help providing a more dynamic scenario of stress induced alterations in murine models. Nonetheless, the feasibility of such procedure still has to be investigated carefully, as the effects of repeated trimming, under general anesthesia, may represent a confounding factor itself and may impair welfare of the animals.

## 5. Conclusions

In conclusion, the study further confirms and strengthens the use of hair as a good matrix for “long-term” endocrinologic evaluations and provides new insights on corticosterone, testosterone and DHEA levels in laboratory male mice. The highlighted effect of age on hormones levels indicate the need for accurate age-related reference intervals, while the higher levels of DHEA detected in grouped animals seem to suggest that such housing practice may be beneficial for social interactions. Overall, despite further studies being needed, it seems that hair hormones quantification may be a good tool for welfare assessment in laboratory mice and may help in refining laboratory animals husbandry practices ameliorating life quality.

## Figures and Tables

**Table 1:** Spearman rank correlation matrix.

	Corticosterone	Testosterone	DHEA	Age
**Corticosterone**	–	−0.587 *p* < 0.0001	−0.479 *p* = 0.0003	−0.604 *p* < 0.0001
**Testosterone**	–	–	0.855 *p* < 0.0001	0.451 *p* = 0.0006
**DHEA**	–	–	–	0.354 *p* = 0.0086
**Age**	–	–	–	–

DHEA = dehydroepiandrosterone.

**Table 2:** Results of linear regression models.

Models	*R* ^2^		Angular Coefficient (β)	95% C.I.
(log)Corticosterone	0.41	Intercept	2.9472	2.8491; 3.0452
		Age	−0.0018	−0.0014; −0.0007
Testosterone	0.24	Intercept	3.8278	2.4543; 5.2013
		Age	0.0103	0.0052; 0.0155
(log)DHEA	0.14	Intercept	1.2245	1.1330; 1.3160
		Age	0.0005	0.0001; 0.0008
